# Draft genome sequence of the glasshouse-potato aphid *Aulacorthum solani*

**DOI:** 10.1093/g3journal/jkaf013

**Published:** 2025-01-24

**Authors:** Joseph Torres, Paula Rozo-Lopez, William Brewer, Omid Saleh Ziabari, Benjamin J Parker

**Affiliations:** Department of Microbiology, University of Tennessee, Knoxville, Knoxville, TN 37916, USA; Department of Biology, The University of North Carolina at Chapel Hill, Chapel Hill, NC 27514, USA; Department of Microbiology, University of Tennessee, Knoxville, Knoxville, TN 37916, USA; Department of Microbiology, University of Tennessee, Knoxville, Knoxville, TN 37916, USA; Department of Biological Sciences, University of Pittsburgh, Pittsburgh, PA 15260, USA; Department of Microbiology, University of Tennessee, Knoxville, Knoxville, TN 37916, USA; Department of Biology, The University of North Carolina at Chapel Hill, Chapel Hill, NC 27514, USA

**Keywords:** *Aulacorthum solani*, genome, de novo assembly, insect, aphid

## Abstract

*Aulacorthum solani* is a worldwide agricultural pest aphid capable of feeding on a wide range of host plants. This insect is a vector of plant viruses and causes injury to crops including stunted growth from the loss of phloem. We found that the publicly available genome for *A. solani* is contaminated with another aphid species, and we produced a new genome using a barcoded isogenic laboratory line. We generated Oxford Nanopore and Illumina reads to assemble a draft genome, and we sequenced RNA to aid in the annotation of our assembly. Our *A. solani* genome is 671 Mb containing 7,020 contigs with an N50 length of 196 kb with a BUSCO completeness of 98.6%. Out of the 24,981 genes predicted by EGAPx, 22,804 were annotated with putative functions based on homology to other aphid species. This genome will provide a useful resource for the community of researchers studying aphids from agricultural and genomic perspectives.

## Introduction

The glasshouse-potato aphid, *Aulacorthum solani* (Kaltenbach, 1843) (Hemiptera: Aphididae; NCBI Taxonomy ID: 202456; also referred to as the foxglove aphid), is a polyphagous species with one of the broadest host plant ranges of any aphid. This species is an agricultural pest of crops such as tomatoes, peppers, tobacco, cucurbits, and legumes (Blackman and Eastwood 1984). It is a particularly important pest of greenhouse crops in North America and the United Kingdom (Jandricic *et al*. 2014). In addition to direct feeding injury that causes discoloration and deformation of leaves, *A. solani* can also vector several plant viruses (Stone *et al*. 2024).

More broadly, aphids are important study organisms for addressing questions in evolutionary biology, developmental biology, and microbial symbiosis (Brisson and Stern 2006; Srinivasan and Brisson 2012). However, only a small percentage of the 5,000 estimated aphid species have available whole genome sequences in NCBI and/or AphidBase (Shigenobu and Yorimoto 2022). *Aulacorthum solani* sits within the tribe Macrosiphini along with key species including the model pea aphid *Acyrthosiphon pisum* (Harris, 1776) (Choi *et al*. 2018), making it a critical species for comparative studies.

We uncovered issues with the previously published reference genome for *A. solani* (NCBI accession ASM852887v1) (Tiwari *et al*. 2021). We show that this assembly is likely contaminated, and that some or all of the genome comes from another aphid species. This misidentification is leading to issues in the literature. For example, a recent attempt to resolve the aphid phylogeny based on ultraconserved elements used the misidentified ASM852887v1 genome. This led to an incorrect placement of *A. solani* in the aphid phylogeny (Liu *et al*. 2024), complicating taxonomic and evolutionary studies of aphids.

Here we describe a high-quality genome assembly for *A. solani* using an isogenic laboratory line. We generated a draft genome using Oxford Nanopore and Illumina reads, and sequenced RNA to aid in genome annotation. Our draft genome is 671 Mb containing 7,020 contigs with an N50 length of 196 kb, and BUSCO scores and other analyses suggest the genome is highly complete. Our annotation contains 24,981 genes, and we use this to compare *A. solani* to the closely related *A. pisum*. Our assembly will serve as a useful reference for the field and will contribute to the use of aphids as models for questions in symbiosis, plant–herbivore interactions, phenotypic plasticity, and agriculture (Brisson and Stern 2006).

## Materials and methods

### Aphid collection and colonization

We collected asexual winged and wingless female *A. solani* adults from garden tomato (*Solanum lycopersicum*) plants in Knoxville, Tennessee, United States, in May 2021. To establish a colony of *A. solani* in the laboratory, we used a single asexual female. After colonization, we maintained this line on fava bean plants at 20°C 16L:8D. To validate our taxonomic identification, we used COI barcoding (LCO1490 5′-GGTCAACAAATCATAAAGATATTGG-3′ and HCO2198 5′-TAAACTTCAGGGTGACCAAAAAATCA-3′), Sanger sequencing, and comparisons of our COI sequence to the Barcode of Life Data System (Foottit *et al*. 2008). COI barcoding is the use of the Cytochrome Oxidase 1 mitochondrial sequence to identify a species that has been cataloged on the BOLD Systems website. The BOLD Systems database allows animal identification (https://v3.boldsystems.org/index.php/IDS_OpenIdEngine) and makes species matches using neighbor-joining trees constructed from the top hits to the COI sequence. Our partial COI barcode sequence was uploaded to NCBI with accession number PQ361290.

### Species ID for the existing *A. solani* assembly

We analyzed the COI sequence found in the putative *A. solani* aphid assembly (accession ASM852887v1) in order to troubleshoot problems with this genome. We downloaded *A. pisum* COI accessions from the Barcoding Life Database (record ID “ACEA116-14”) and used the sequences to BLAST the genome assembly. We then took the top alignment that spanned the entire region and queried it in the Barcoding Life Database as above.

### DNA extraction and sequencing

We pooled 7 genetically identical adult unwinged aphids cultivated in the laboratory and isolated genomic DNA (gDNA) using Bender buffer and ethanol precipitation (Bender *et al*. 1983). We then sheared the gDNA to approximately 20 kb fragments using Covaris G-tubes (Covaris LLC., Woburn, Massachusetts, United States) at 4200 RMP for 1 min, followed by tube inversion. Part of the sample was set aside for Illumina sequencing. For Oxford Nanopore library preparation, we used the NEB Next PPFE repair kit with Ultra II end prep reaction (New England Biolabs, Ipswich, Massachusetts, United States) under recommended conditions and Nanopore ligation sequencing kit SQK-LSK110. For sequencing, we used a Nanopore R9.4.1 (FLO-MIN106D) flow cell and a MinION MIN-101B sequencing device (Oxford Nanopore Technologies, Oxford, United Kingdom). We ran the flow cell for 24 h, followed by a wash with Flow Cell Wash Kit (EXP-WSH004); we then reloaded the flow cell with a second library prep and ran the sequencer for an additional 48 h. We stopped the second sequencing run at 24 h (∼11 Gb of sequencing). Raw nanopore sequencing reads are available in the NCBI Sequence Read Archive under BioProject ID PRJNA1156622 with BioSample accession SAMN43496284 and run ID SRR30661341.

In addition, we conducted Illumina sequencing of gDNA at Novogene (Novogene Corporation Inc., Sacramento, California, United States) using DNA extracted as above. We generated 15.5 billion base pairs with 150 bp paired-end reads on an Illumina NovaSeq platform. Illumina reads are available in the NCBI Sequence Read Archive with BioSample accession SAMN43799130 and run ID SRR30686038.

### RNA extraction and sequencing

We homogenized pools of 5 genetically identical unwinged adult aphids with a pestle in 800 µL of TRIzol (Invitrogen; Thermo Fisher Scientific, Inc., Waltham, Massachusetts, United States) and extracted total RNA using chloroform and isopropanol precipitation. We used the Zymo RNA Clean & Concentrator kit (Zymo Genetics Inc., Seattle, Washington, United States) to improve the purity and to remove DNA using DNAse I. We then performed transcriptome sequencing at Novogene (Novogene Corporation Inc., Sacramento, California, United States). Library preparation was conducted using poly-A selection. The libraries were sequenced to approximately 3.4 billion base pairs (bp) per sample with 150 bp paired-end reads on an Illumina NovaSeq platform. Raw reads were deposited into the NCBI Sequence Read Archive under BioProject ID SRP532563 with BioSample accession SAMN43784434 and run ID SRR30675520.

### *Aulacorthum solani* whole genome assembly

We used Guppy (Oxford Nanopore Technologies) for base calling and quality trimming raw reads (Wick *et al*. 2019). We assembled Oxford nanopore reads using Canu v.2.0 with recommended parameters and a predicted genome length of 300 Mb (Koren *et al*. 2017). We then mapped Illumina reads to the assembled contigs using BWA (Li and Durbin 2010). We used these alignments to polish assembled contigs with Pilon for 2 rounds (Walker *et al*. 2014) and then assessed with BUSCO v.5.7.0 using Hemiptera as the BUSCO lineage parameter (Manni *et al*. 2021). We removed haplotigs with PurgeHaplotigs by mapping the contigs against the original Oxford nanopore reads and selecting the appropriate valley points as shown in the coverage histogram (Supplementary Fig. 1) (Roach *et al*. 2018).

### Contig purging

We removed all contigs that matched a phylum other than Arthropoda, including from Proteobacteria, using the Blobtools visualizer after BLAST searching the contigs against the NCBI nucleotide NR database with the BLASTplus command line interface (Supplementary Fig. 2) (Camacho *et al*. 2009; Challis *et al*. 2020). In addition, we manually removed 2,058 contigs based on mapped Illumina read depth and an average short read coverage below 14x, as the SD was higher than the average coverage. The remaining contigs were assessed again with BUSCO. The *A. solani* genome is available in NCBI with BioProject ID PRJNA1156622 accession JBHJKM000000000.

### *Aulacorthum solani* genome annotation

We used RNA-seq data to annotate the assembled contigs using NCBI's alpha-phase EGAPx v.0.2 annotation pipeline. We used the resulting gene predictions for automated functional annotation with the EnTAP pipeline using the default eggNOG-mapper with homology searches against the NCBI protein NR database, the UniProt Swiss-Prot database, the UniProt Tremble database, and the ref-seq invertebrate database with bacteria set as the contaminant flag (Hart *et al*. 2020). We then submitted the predicted genes to BLAST-koala to obtain up-to-date KEGG ortholog terms (Kanehisa *et al*. 2016). We compared the pathway modules to the pea aphid *A. pisum* AL4f, Project ID PRJNA547584, BioSample SAMN10253041. The individual commands and slurm scripts as well as the KEGG assignments are available on GitHub (https://github.com/Artifice120/Aulacorthum_solani_genome_assembly_pipeline/tree/main).

## Results

### Analysis of the existing *A. solani* assembly

BLAST results of the *A. pisum* COI sequence from the BOLD database against the ASM852887v1 genome assembly hit the PVMI01058906.1 scaffold from base pairs 9069-9726 with a 92.9% identity. We found that this region of the genome matched *Brachycaudus helichrysi* as a top hit with a 100% match to the BIN barcode in BOLD systems. For our genome, we only used aphids from an isogenic colony (AS13) that has been maintained in the laboratory for multiple generations and confirmed the species identification by COI barcoding. Furthermore, after genome assembly, we reconfirmed the presence of the COX1 barcode identification for *A. solani* in contig tig00020064 (9727-10437) with a 100% match to the *A. solani* BIN barcode in BOLD Systems.

### *Aulacorthum solani* whole genome assembly

**Fig. 1.**
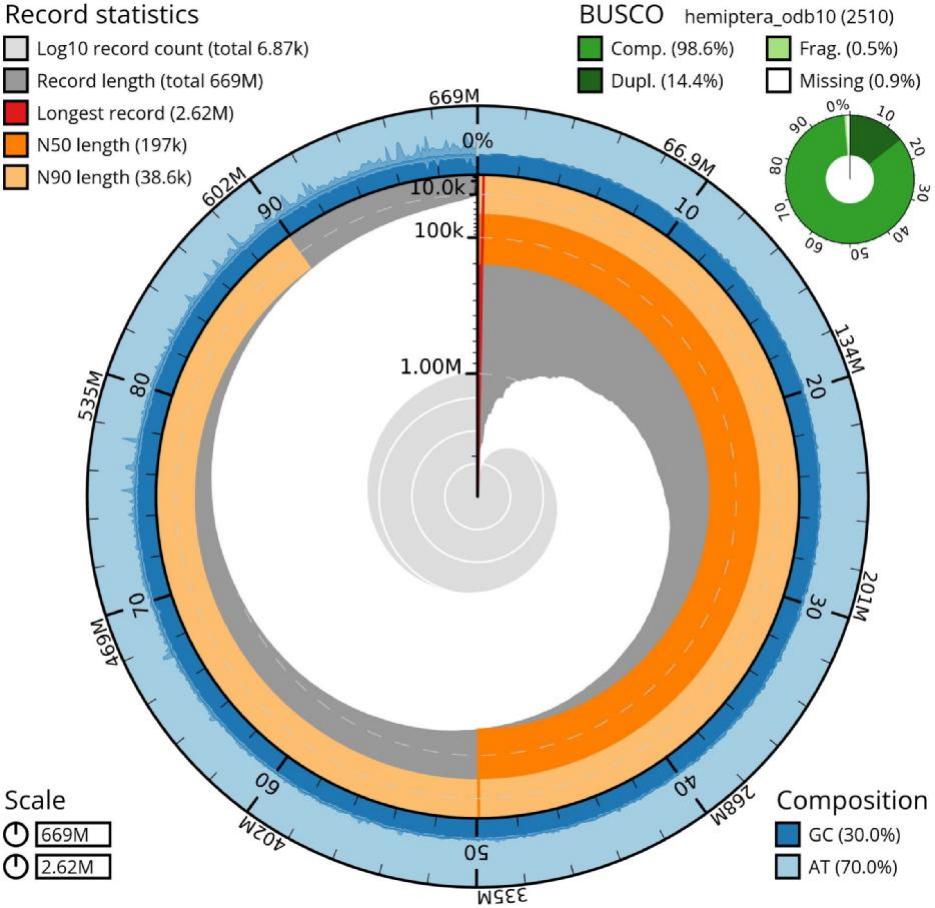
Snail plot summary of assembly statistics for *A. solani* assembly. The main plot is divided into 1,000 size-ordered bins around the circumference of the snail plot with each bin representing 0.1% of the 670,586,136 bp assembly. The distribution of the sequence lengths is also shown in dark gray with the plot radius scaled to the longest sequence in the assembly (2,615,422 bp). The orange and pale orange arcs represent the N50 (195,973 bp) and N90 (38,241 bp) sequence lengths, respectively. The pale gray spiral shows the cumulative sequence count on a log scale with the white dotted lines showing successive orders of magnitude. The pale blue area on the outer edge of the plot represents the distribution of the GC and AT percentages. A summary of the complete, fragmented, and duplicated BUSCO genes in the hemiptera_od10 set is shown in the top right corner.

We obtained a total of 4,873,637 nanopore reads (at an average length of 6.7 kb) and 10,344,638 Illumina reads (PE 150 bp). After assembly, haplotig purging, polishing, removal of plant and bacterial contigs, and contig bubble resolution, our assembly contained 7,020 contigs with an N50 length of 196 kb and a total length of 671 Mb. The assembly has a BUSCO completeness score of 98.6% (*n* = 2,510) and a duplication score of 14.4% (Fig. 1). The genome size is slightly larger than closely related species including *A. pisum* (514.2 Mb) but within the range of aphid genomes (317.1–711.3 Mb estimated by flow cytometry; Wenger *et al*. 2017). GC content at 30.0% was similar to other aphid species [*A. pisum*: 29.6% (International Aphid Genomics Consortium 2010); *Myzus persicae*: 30.1% (Singh *et al*. 2021)].

In addition to contigs with homology to the aphid obligate symbiont *Buchnera aphidicola*, we removed contigs with homology to the Alphaproteobacteria *Wolbachia*, suggesting that our aphid line is infected with *Wolbachia*. Analysis of these contigs yielded a BUSCO score of 37.3% complete using the Rickettsiales_odb10 database, suggesting the microbes were not at high enough density in our aphid sample to produce a useful genome assembly for this bacterium under these conditions.

### *Aulacorthum solani* genome annotation

A total of 24,981 genes were predicted by EGAPx based on protein alignments and HMM models appropriate to the taxonomy as well as RNA sequencing data. We found 93.4% of functional annotation assignments are within the Arthropoda lineage, with the highest homology toward *A. pisum* (pea aphid) (Supplementary Fig. 3). This was visualized further with a synteny plot showing the mapped regions between the *A. pisum* reference genome and this *A. solani* draft genome (Supplementary Fig. 4). Many of the annotated genes had putative functions such as detoxification genes, salivary genes, virus transmission genes, transcription factor genes, and mitochondrial genes (Table 1), which are also found in other aphid species such as *A. pisum*, *Metopolophium dirhodum*, *M. persicae*, *Aphis craccivora*, and *Aphis glycines*. Out of the 24,981 genes predicted by EGAPx, 15,332 were annotated with families that were different from *A. pisum* with 22,780 annotated genes total from the aphid superfamily.

**Table 1.** Number of predicted *A. solani* genes with putative functions based on the reference protein databases [NCBI BLASTX “Nr” (nonredundant), UniProt Swiss-Prot, UniProt tremble, and the ref-seq invertebrate database].

i.	Defensive and detoxification genes	
a)	Cytochrome P450	98
b)	Glutathione S-transferase	29
c)	ABC transporter	66
d)	Carboxyl/cholinesterase	41
e)	UDP-glucosyltransferase/UDP-glucuronosyltransferase	123
f)	Cuticle protein	91
ii.	Salivary genes and chemoreceptors	
a)	Glucose dehydrogenase	36
b)	Sugar/glucose/inositol transporter	41
c)	Apolipophorin	2
d)	Salivary gland/protein/peptide	8
e)	Secretory protein	10
a)	Gustatory receptor	215
b)	Odorant receptor/protein	124
iii.	Insecticide resistance genes	
a)	Acetylcholine receptor/esterase/transporter	21
b)	Sodium exchanger/symport/channel/neurotransmitter/transporter	100
c)	GABA transporter	2
d)	Multidrug resistance protein	30
iv.	Virus transmission genes	
a)	Dynamin	37
b)	Serine protease inhibitor	12
c)	Vesicle transport/trafficking	10
d)	Endocytosis	8
e)	Exocytosis	4
v.	Transcription factors	379
vi.	Mitochondrial genes	599

In addition, we found 98 total cytochrome P450 oxidase genes that were distributed across 30 families (Supplementary Fig. 5).

### Differences with the *A. pisum* genome

We identified several potentially significant differences between the *A. solani* and *A. pisum* genomes. First, *A. solani* appears to have an expanded number of odorant receptors, with 17 different odorant receptor families present in our annotation compared with 9 in the *A. pisum* genome. Next, KEGG annotations highlighted 3 complete pathway modules that are present in *A. solani* that are missing from *A. pisum* (M00131, M00132, and M00415) (Kanehisa *et al*. 2016). Specifically, M00131 and M00132 are part of inositol phosphate metabolism, with the first incorporating myo-inositol and the second incorporating phytate into inositol phosphate metabolism. The pathway module M00415 corresponds to fatty acid elongation in the endoplasmic reticulum while the *A. pisum* KEGG annotations only show complete modules with fatty acid elongation in the mitochondria.

## Discussion

We generated an accurate draft genome assembly for *A. solani*. Our analysis of complete BUSCOs (98.6%) suggests that our *A. solani* draft genome assembly is highly complete. We found evidence of infection of this aphid with *Wolbachia* though we could not assemble a genome for this symbiont from our data, but this result does add to the growing number of aphid species that have been identified as hosting *Wolbachia* suggesting it is more common among aphids than was previously acknowledged. Future work to reduce duplication in the genome and chromosome-scale genome scaffolding would improve this genome assembly, and additional coverage could produce sequences for *Wolbachia* and any other microbial associates of this aphid.

A key difference between *A. solani* and the model pea aphid *A. pisum* appears to be the incorporation of myo-inositol and phytate into inositol phosphate metabolism. *Aulacorthum pisum* has been shown to not absorb myo-inositol or its free cyclitol form (phytic acid); the concentration of cyclitols in honeydew from *A. pisum* is higher than the concentration in plant stems and leaves (Campbell and Binder 1984). An intriguing possibility is that these metabolic differences contribute to the broad host range of *A. solani*, which could be consistent with findings from other generalist invertebrate species (Sanni *et al*. 2017). Host plant range differences could also be linked to the expanded repertoire of odorant receptors found in *A. solani*. Future work could focus on the genomic basis of herbivore generalism given the broad host range of this insect.

Our work also corrects the record with respect to the existing ASM852887v1 assembly for *A. solani*. BLAST results suggest this assembly was either misidentified or was contaminated by multiple aphid species, including *B. helichrysi*. We ensured our barcode-confirmed *A. solani* isogenic line had no contamination from other species at every step of the genome assembly process. Identification of aphids is difficult as many species exhibit significant similarities in their morphology. Misidentification is often compounded by phenotypic variation within a single species, making accurate identification challenging without specialized mounting techniques or molecular methods like DNA barcoding. In addition, multiple species can be found on the same host plant species, and there is a need to carefully colonize isogenic lines from single parthenogenic aphids in the laboratory before generating sequencing data originated from pooling individuals. The genus *Aulacorthum* is especially challenging since there are likely cryptic species groups, species placements shared with *Acyrthosiphon*, and poor coverage of barcode reference sequences for nonagriculturally relevant species.

Along with quality assemblies from a growing number of aphid species, the *A. solani* draft genome provides a valuable tool for studying insect diversification and evolution, plant–insect interactions, and aphid–virus interactions. Future work using this genome will increase the utility of aphid systems for addressing basic biological questions and could have practical application in the control and management of this pest species.

## Supplementary Material

jkaf013_Supplementary_Data

## Data Availability

This project can be found under NCBI BioProject ID PRJNA1156622. Raw nanopore sequencing reads are available in the NCBI Sequence Read Archive (SRA) with BioSample accession SAMN43496284 and run ID SRR30661341. Genomic Illumina reads are available in the SRA with BioSample accession SAMN43799130 and run ID SRR30686038. RNA-seq reads were deposited into SRA under BioProject ID SRP532563 with BioSample accession SAMN43784434 and run ID SRR30675520. The partial COI barcode sequence for the sequenced line was uploaded to NCBI with accession number PQ361290. The *A. solani* genome is available in NCBI with accession JBHJKM000000000. The individual commands and slurm scripts as well as the KEGG assignments are available on GitHub (https://github.com/Artifice120/Aulacorthum_solani_genome_assembly_pipeline/tree/main). Supplemental material available at G3 online.
