# Supplementary material for: Draft genome sequence of the glasshouse-potato aphid Aulacorthum solani
Source: G3 (Bethesda). 2025 Jan 24;15(3):jkaf013. doi: 10.1093/g3journal/jkaf013 (PMC11917484; doi:10.1093/g3journal/jkaf013)
Supplement: jkaf013_Supplementary_Data [file jkaf013_supplementary_data.pdf]

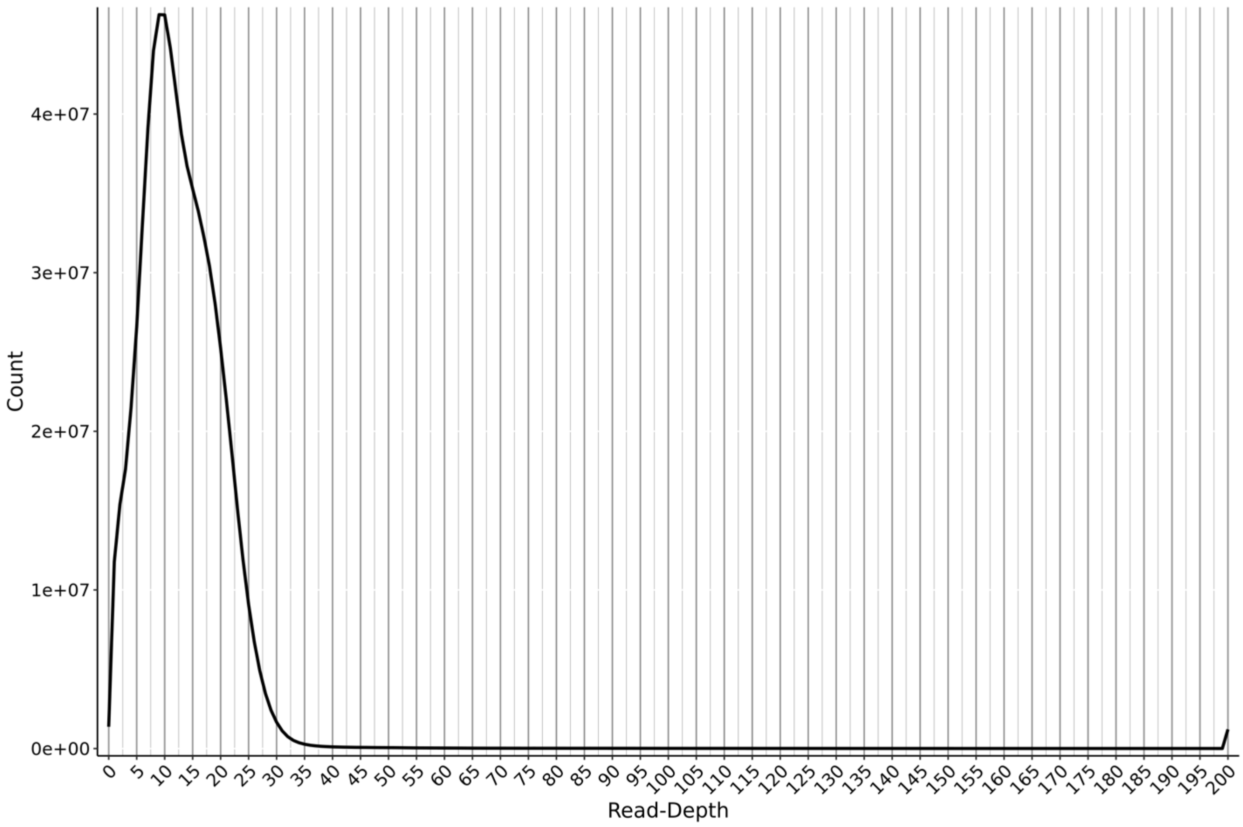

Figure S1: Genome-wide read depth histogram with overlapping Bimodal distribution. A low cutoff of 5, a midpoint of 15, and a high cutoff of 60 were selected in this instance.

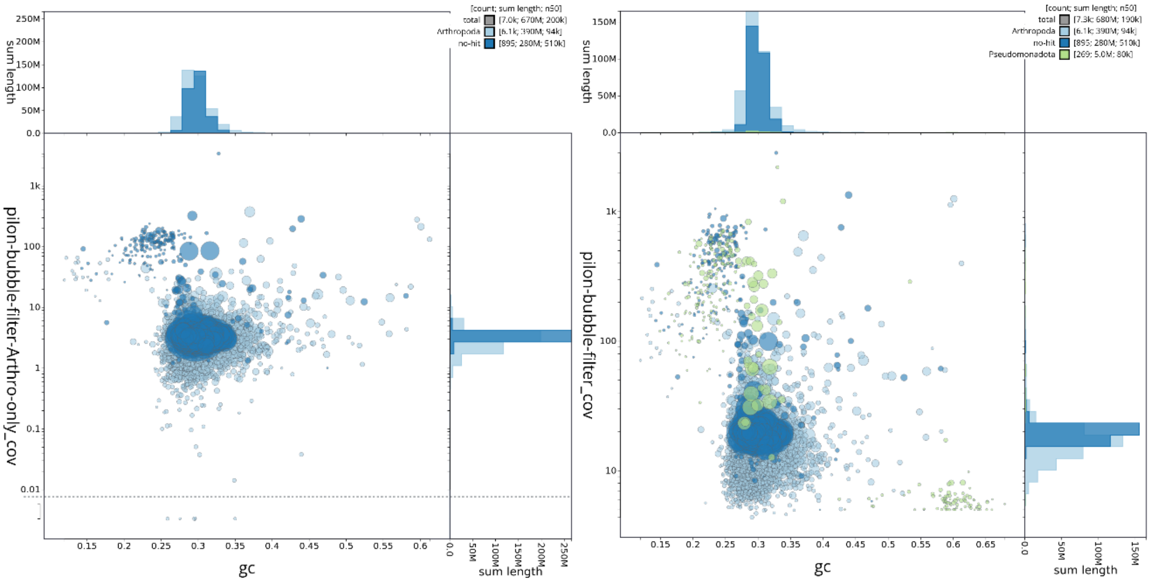

Figure S2. Blob plot of base coverage of the final *A. solani* assembly mapped against Canu reads (bam file called pilon-bubble-filter-Arthro-only_cov) plotted against GC proportion for sequences in the *A. solani* assembly. Sequences are colored by Phylum. Circles are sized in proportion to sequence length on a square-root scale, ranging from 1,129 to 2,615,422. Histograms show the distribution of sequence length sum along each axis. The left plot is after the removal of contaminating Pseudomonadota lineage contigs. Contigs without BLAST taxonomy hits were retained.

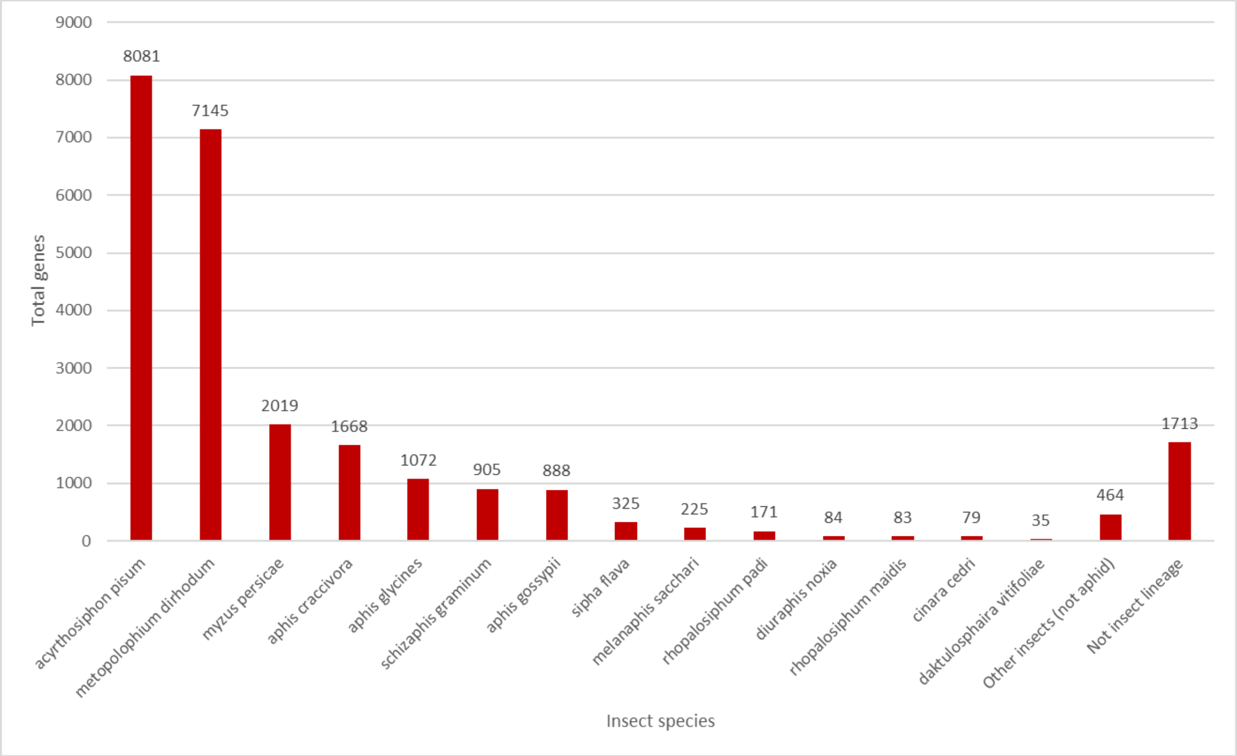

Figure S3. Gene annotation summary of *A. solani* draft genome (n=25,000) organized by major aphid species based on the protein databases (NCBI BlastX ‘nr’ (non-redundant), Uniprot Swissprot, UniProt tremble, and the ref-seq invertebrate database).

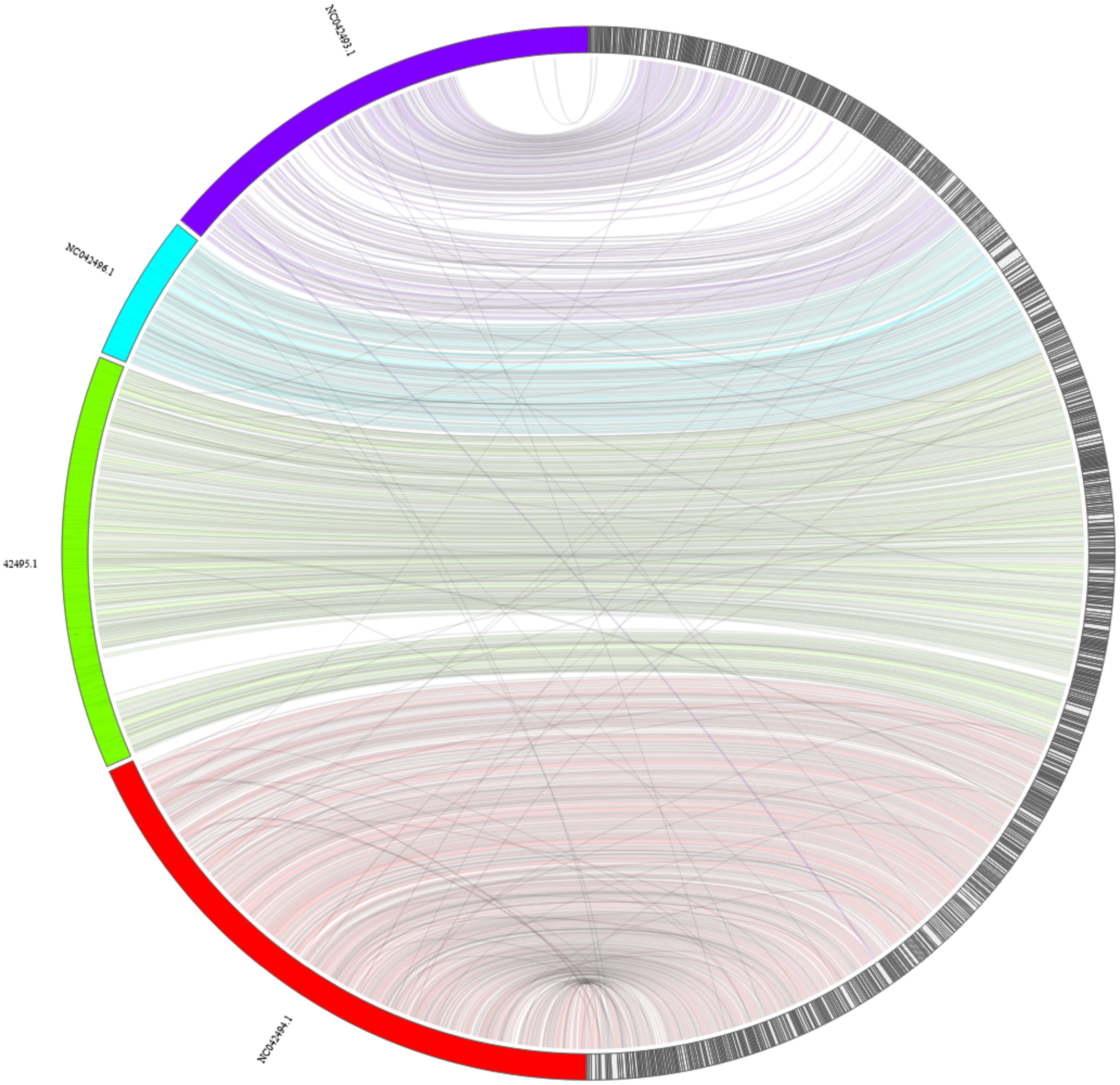

Figure S4: Ortholog mapping between *Acyrthosiphon pisum* chromosomes and *Aulacorthum solani* contigs.

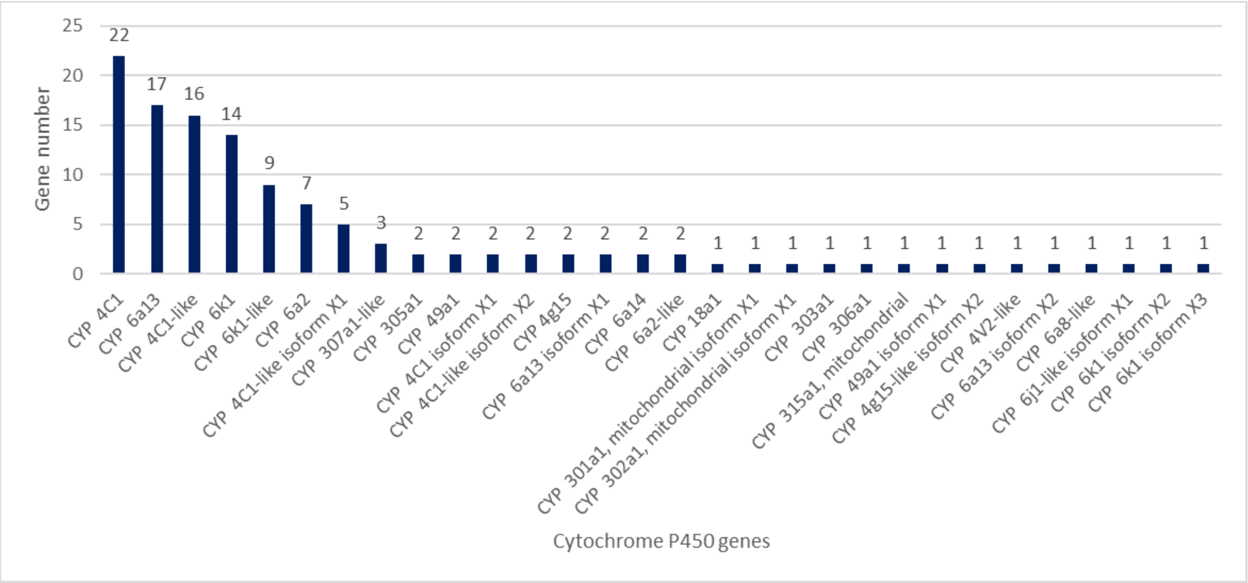

Figure S5. Distribution of the detoxification enzyme cytochrome P450 (CYP) genes (n=123) annotated in the *Aulacorthum solani* draft genome.
